# Molecular Characterization of Highly Pathogenic H5N1 Avian Influenza A Viruses Isolated from Raccoon Dogs in China

**DOI:** 10.1371/journal.pone.0004682

**Published:** 2009-03-09

**Authors:** Xian Qi, Xihan Li, Paul Rider, Weixing Fan, Hongwei Gu, Longtao Xu, Yonghua Yang, Sangwei Lu, Hua Wang, Fenyong Liu

**Affiliations:** 1 Jiangsu CDC-Nanjing University Joint Institute of Virology, State Key Laboratory of Pharmaceutical Biotechnology, School of Life Sciences, Nanjing University, Nanjing, Jiangsu, China; 2 Jiangsu Center for Disease Control and Pevention, Nanjing, Jiangsu, China; 3 Division of Infectious Diseases, School of Public Health, University of California, Berkeley, California, United States of America; 4 China Animal Health and Epidemiology Center, Qingdao, Shandong, China; 5 Qilu Animal Health Products Co. Ltd, Jinan, Shandong, China; Institute of Molecular and Cell Biology, Singapore

## Abstract

**Background:**

The highly pathogenic avian influenza H5N1 virus can infect a variety of animals and continually poses a threat to animal and human health. While many genotypes of H5N1 virus can be found in chicken, few are associated with the infection of mammals. Characterization of the genotypes of viral strains in animal populations is important to understand the distribution of different viral strains in various hosts. This also facilitates the surveillance and detection of possible emergence of highly pathogenic strains of specific genotypes from unknown hosts or hosts that have not been previously reported to carry these genotypes.

**Methodology/Principal Findings:**

Two H5N1 isolates were obtained from lung samples of two raccoon dogs that had died from respiratory disease in China. Pathogenicity experiments showed that the isolates were highly pathogenic to chicken. To characterize the genotypes of these viruses, their genomic sequences were determined and analyzed. The genetic contents of these isolates are virtually identical and they may come from the same progenitor virus. Phylogenetic analysis indicated that the isolates were genetically closely related to genotype V H5N1 virus, which was first isolated in China in 2003, and were distinct from the dominant virus genotypes (e.g. genotype Z) of recent years. The isolates also contain a multibasic amino acid motif at their HA cleavage sites and have an E residue at position 627 of the PB2 protein similar to the previously-identified avian viruses.

**Conclusions/Significance:**

This is the first report that genotype V H5N1 virus is found to be associated with a mammalian host. Our results strongly suggest that genotype V H5N1 virus has the ability to cross species barriers to infect mammalian animals. These findings further highlight the risk that avian influenza H5N1 virus poses to mammals and humans, which may be infected by specific genotypes that are not known to infect these hosts.

## Introduction

Influenza A viruses are clinically and/or economically important pathogens in a variety of animals, including humans, pigs, horses, marine mammals, and poultry [1], [2]. They are classified into different subtypes on the basis of antigenic properties in the two surface glycoproteins, the haemagglutinin (HA) and neuraminidase (NA). Wild aquatic birds are the primary natural reservoir of influenza A viruses, which harbor all currently known 16 HA and 9 NA subtypes [3]. There are limited numbers of host-specific influenza A viruses, i.e., humans (H1N1, H3N2, H2N2, and H1N2), pigs (H1N1, H3N2 and H1N2), horses (H3N8 and H7N7), and domestic avian species (H5N1, H5N2, H7N7, and H9N2) [1]. All known lineages of influenza A viruses in mammals and domestic avian species originate from the virus pool of wild aquatic birds. Depending on their ability to cause disease in chickens, avian influenza viruses (AIVs) are characterized as low pathogenic (LP) or highly pathogenic (HP). AIVs in their natural reservoirs are in evolutionary stasis. They are usually nonpathogenic but may become highly pathogenic to chickens through mutations after introduction from wild aquatic birds. Subtypes H5 and H7 avian influenza viruses have become highly pathogenic to terrestrial poultry (chickens and turkeys), mainly due to mutations resulting in multiple basic amino acids in the connecting peptide between the HA1 and HA2 domains of the HA0 precursor protein [1]. It had been believed that avian influenza virus could not cross the species barrier to directly infect humans, and that pigs are required as an intermediate host for the interspecies transmission of avian influenza viruses to humans or as mixing vessels for the generation of reassortant viruses [2]. However, it was reported in 1997 that 18 people became infected with HPAIV H5N1 and 6 died [1], [4], [5]. As of now, only AIVs H5N1, H7N7, H7N3, and H9N2 have been reported to have the ability to directly transmit to humans and may cause human disease [1], [4]. However, whether specific genotypes of HPAIV H5N1 (e.g. genotype V) can cross the species barrier and infect mammals other than humans has not been extensively investigated.

HPAI H5N1 viruses have become endemic in poultry in southern China, and have posed a serious threat to wildlife and human health since 1997 [4], [6]–[10]. As of April 2008, a total of 381 human cases of H5N1 infection have been recorded, including 240 deaths (WHO, 2008). The HPAI H5N1 virus was first detected in diseased geese in southern China in 1996. In the following years, due to the virus rapid evolution by genetic drift and genetic shift, a diversity of H5N1 genotypes were isolated and multiple sublineages were established in southern China [6], [8]. In 2002, H5N1 viruses, which are typically not lethal to ducks, were observed to be highly pathogenic to wild aquatic birds in Hong Kong [11]. From late 2003, the H5N1 viruses expanded their geographical range, and resulted in unprecedented epizoonosis in poultry in eastern and southeastern Asia. Since the HPVI H5N1 viruses caused the first large-scale outbreak in migratory waterfowls at Qinghai Lake (QH) in western China in May 2005 [7], QH-like H5N1 viruses have rapidly spread to Europe and Africa, which possibly resulted from the migration of wild birds [1], [7], [12], [13]. Since late 2005, the Fujian-like H5N1 avian influenza virus has predominated in southern China [10]. In addition to humans, HPAIV H5N1 viruses naturally infect a range of mammalian species such as pigs, tigers, leopards, domestic cats, viverrids, stone marten and dogs [1], [13]–[22]. Furthermore, H5N1 virus infections in the laboratory have been documented in mice, ferrets, monkeys, pigs, and cats [23]–[28].

In this study, we isolated two H5N1 viruses from raccoon dogs suffering from respiratory disease on a farm in eastern China in 2005, and studied their genotype and pathogenicity in chickens. Our studies revealed that the raccoon dog isolates had each of the eight gene segments of avian origin, were highly pathogenic to chickens, and genetically belonged to H5N1 genotype V viruses. The recovery of H5N1 viruses from dead raccoon dogs underlines the importance of enforcing H5N1 virus surveillance in mammals, especially carnivorous animals, in the epidemiology of a potential HPAIV outbreak.

## Results

### Isolation and identification of avian influenza viruses and investigation of their pathogenicity in chickens

Two virus samples with hemagglutination activity were isolated from the lung samples taken from each of the two dead raccoon dogs using embryonic eggs, and were designated as A/Raccoon dog/Shandong/sd1/2005 and A/Raccoon dog/Shandong/sd2/2005, respectively. No bacteria were isolated from the specimens. The isolates were determined to be H5N1 subtype by HI and NI assays and were further characterized by genomic sequencing and the nucleotide BLASTn analysis. The pathogenicity of the isolates was evaluated in eight 5-week-old specific-pathogen-free (SPF) chickens by intravenous inoculation. Both viruses gave rise to death in all 8 chickens within 24 hours, and therefore according to the World Organization for Animal Health criteria, the intravenous virus pathogenicity index (IVPI) was 3 and the mean death time (MDT) was 1 day. Thus, both viruses were highly pathogenic to chickens.

### Genotype identification and phylogenetic analysis of the isolated viral strains

To investigate the genotype and genetic origin of the isolates, we generated cDNA clones from the genomes of these viruses using reverse transcription-PCR (RT-PCR) amplification [29]. We subsequently determined the nucleotide sequence of the complete genome of the two viruses isolated from the raccoon dogs, each of which contains 8 genes. The nucleotide sequences obtained in this study have been deposited in the GenBank database (accession numbers EU420038–EU420053). The percentage of nucleotide sequence homology among the eight gene segments of the two isolates ranged between 99.9% and 100%, and the predicted amino acid sequence homology is 100%. Thus, their genetic characterizations were virtually identical, suggesting that the two isolates originated from the same progenitor viruses. The genotype and genetic origin of the isolates were initially inferred from BLAST analysis and pair wise comparisons of each gene segment to the corresponding sequences of reference viruses. The results indicated that the 8 gene segments from the isolates share the highest sequence homology with other H5N1 viruses circulating in China and Japan in 2003–2005 (>99%) (Table 1). Since 2002, diverse genotypes of H5N1 virus have been found in southern China and southeast Asia, including V, W, X, Y, Z+ and Z [6], [8]. In our study, the two isolates share the highest homology (98.1–99.8%) of all 8 gene segments with reference viruses such as A/Migratory duck/Jiangxi/2300/2005, A/Chicken/Kyoto/3/2004 and A/Chichen/Shantou/4231/2003, which belong to previously described H5N1 genotype V viruses (Table 1)[6], [8]. These results imply that the raccoon dog isolates belong to genotype V.

**Table 1:** Genetic similarity between RD/SD/sd1/2005 and reference strains available in GenBank.

Gene	Region compared (nt)^a^	Virus with the greatest similarity^b^	Genotype	Similarity (%)
PB2	28–2308	A/migratory duck/Jiangxi/2300/2005	V	99.0
		A/chicken/Kyoto/3/2004	V	99.0
PB1	25–2298	A/migratory duck/Jiangxi/2300/2005	V	98.7
		A/chicken/Kyoto/3/2004	V	98.5
PA	46–2175	A/chicken/Shantou/4231/2003	V	99.0
		A/migratory duck/Jiangxi/2300/2005	V	99.1
HA	29–1735	A/chicken/Shantou/4231/2003	V	98.4
		A/migratory duck/Jiangxi/2300/2005	V	98.1
NP	46–1542	A/Beijing/01/2003	Z	99.0
		A/migratory duck/Jiangxi/2300/2005	V	98.9
NA	21–1370	A/bar-headed goose/Qinghai/0510/05	Z	99.1
		A/migratory duck/Jiangxi/2300/2005	V	99.1
M	26–784	A/Beijing/01/2003	Z	99.8
		A/migratory duck/Jiangxi/2300/2005	V	99.7
NS	45–719	A/chicken/Shantou/4231/2003	V	98.8
		A/goose/Jilin/hb/2003	Z	99.6

a nt, nucleotide.

b The numbers in brackets are GenBank accession numbers for the reference virus.

**Figure 1:**
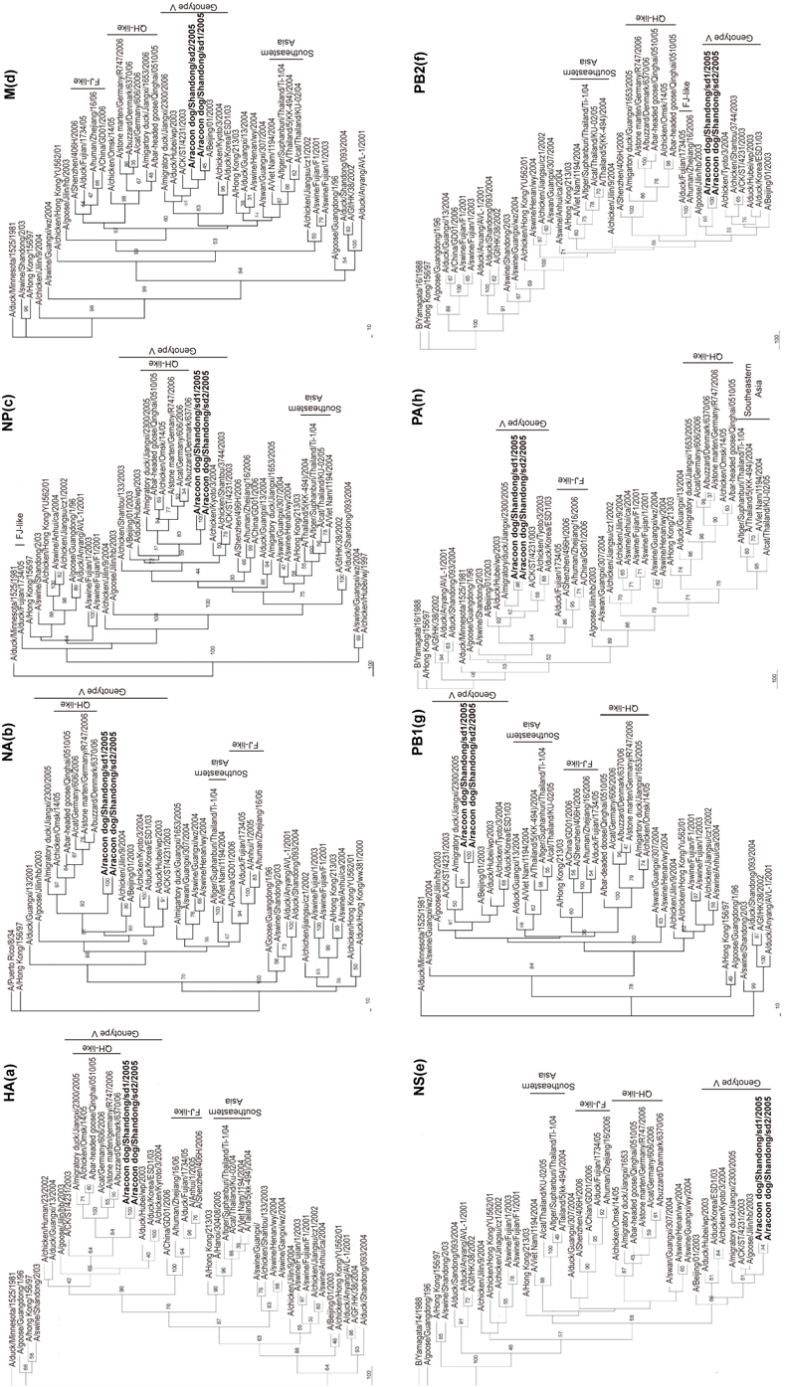
Phylogenetic trees for the HA(a), NA(b), NP(c) , M(d), NS(e), PB2(f), PB1(g) and PA(h) genes of the raccoon dog isolates and related reference viruses. The evolutionary relationships among these viruses were estimated by the method of the neighbor-joining method with 100 bootstraps by using PHYLIP (the PHYLogeny Inference Package) version 3.65. Sequence comparisons to the reference viruses were performed by using the Lasergene software package (DNASTAR, Madison, WI, USA). Alignments of each influenza virus sequence were created using program ClustalX 1.83. The sequence regions compared were as follows: nt 77–1069 (993 bp) of HA, 21–1370 (1350 bp) of NA; 1036–1916 (881 bp) of PB2, 25–1023 (999 bp) of PB1, 1465–2172 (708 bp) of PA, 46–1445 (1400 bp) of NP, 26–785 (760 bp) of M, 27–704 (678 bp) of NS. The trees of HA, PB1,NP and M genes are rooted to A/duck/Minnesota/1525/1981 (H5N1). The trees of PB2, PA, and NS genes are rooted to B/Yamagata/14/1988, and the NA tree is rooted to A/Puerto Rico/8/34 (H1N1).

To characterize more precisely the genetic origin of the gene segments of the raccoon dog viruses, we constructed phylogenetic trees. Reference viruses consisted of H5N1 viruses isolated in poultry, human, and mammals from 1996–2006 (Figure 1). In the HA tree, there are several distinct clades, which show the genetic diversity of H5N1 viruses in recent years in Asia. Raccoon dog viruses belonged to the genotype V group. In contrast, H5N1 viruses isolated from human or swine were grouped into different clades, and viruses from humans in 2005–2006 belonged to the Fujian-like clade in China [11]. The other seven genes of raccoon dog viruses have similar phylogenetic trees with the HA tree. In the HA, NA, and NP trees, QH-like viruses form an independent sublineage clustered with genotype V viruses, and the results suggest that the HA, NA, and NP genes of QH-like viruses could originate from genotype V virus [6], [7]. Thus, phylogenetic analysis of all eight gene segments suggest that the raccoon dog viruses are of avian origins, and they cluster with genotype V viruses (such as A/Migratory duck/Jiangxi/2300/2005, A/Chicken/Kyoto/3/2004, and A/Chichen/Shantou/4231/2003), which are distinct from the dominant H5N1 virus genotype Z associated with poultry outbreaks and transmission to humans in Southern Asia, Europe, and Africa since 2003 [1], [6], [8].

### Molecular characterization of the isolated viral strains

**Figure 2:**
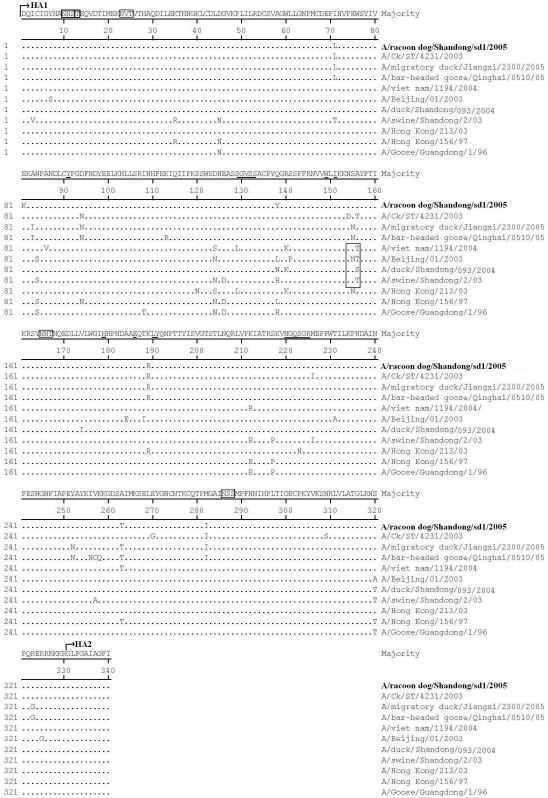
Amino acid sequence comparison of the HA1 proteins between the raccoon dog/Shan dong/sd1/2005 and the reference viruses. Only the amino acids different from those in the consensus sequence are indicated. Numbering starts at the N terminus of HA1. Underlined residues represent the receptor-binding sites. The boxed residues indicate potential glycosylation sites.

Based on the deduced amino acid sequence, the raccoon dog isolates contained the multibasic amino acid motif PQRERRRKKR/GL at their HA cleavage sites, which is a characteristic of highly pathogenic avian influenza viruses [1], [6], [8]. Seven potential N-linked glycosylation sites were found in the HA protein, five of which are located in HA1 (Figure 2). Compared to most H5N1 viruses in China and southeastern Asia in 2002–2004, raccoon dog isolates exhibited a T/S156A mutation, which results in the loss of a glycosylation site [1], [6], [8]. This mutation was also found in QH-like viruses. Amino acid residues related to receptor binding were identical to those A/Goose/Guangdong/1/96-like viruses (Figure 2) [1], [6], [8]. Several studies previously showed that some mutations (such as Q222L, G224S, S223N, N182K, Q192R, L129V, A134V) play a role in enhancing the binding of H5N1 virus to the sialic acid (SA) α2,6-Gal receptor [20], [30]–[34]. These mutations were not found in the HA of raccoon dog isolates, implying that the isolates preferentially bind to α2,3-NeuAcGal linkages of the avian cell receptors rather than α2,6-NeuAcGal linkages of the human cell receptors. Compared with the NA of A/goose/Guangdong/1/96, raccoon dog isolates contain a 20 amino acid deletion (located on resides 49–69) in the stalk of NA molecules, which is identical to the genotypes V and Z [1], [6], [8].

Antivirals are essential for the treatment and prevention of influenza infections. It has been reported that several amino acid mutations, including E119V, R293K, N294S, and H274Y (numbered according to NA of N2 subtype), could confer viral resistance to NA inhibitors (e.g. oseltamivir and zanamivir) [35]. The substitutions 274 (H→Y) and 294 (N→S) were reported to confer resistance to oseltamivir in clinical influenza (H5N1) isolates [36], [37]. No similar amino acid substitutions were observed at the conserved residues in the NA of the raccoon dog viruses. These results suggest that the isolated viruses are sensitive to NA inhibitors. Amantadine and rimantadine target the M2 protein, and single mutations in the trans-membrane domain of M2 (e.g. residue 26 (L→F), 27 (V→A or T), 30 (A→T or V), 31 (S→N or R), and 34 (G→E)) can confer resistance to these drugs [38]. No amino acid substitutions were found at these residues, suggesting that the raccoon dog isolates are sensitive to this class of antiviral drugs.

Several amino acid mutations of the polymerase (PB2, PB1, and PA) and NP protein may have an important effect on the virulence and adaptation of H5N1 virus in the hosts including mice [39]–[42]. Studies have shown that amino acid substitutions (e.g. residue 627 (E→K) and 701 (D→N) of PB2, 13 (L→P), 678 (S→N) of PB1, 615 (N→K) of PA, and 319 (N→K) of NP) may increase the virulence of H5N1 virus in mammals (Table 2) [39]–[42]. Recently, a study revealed that a single amino acid change in PB1 (Y436H) and PA (T515A) reduced the viral lethality in ducks inoculated by natural routes [43]. Sequence analysis revealed amino acid residues P13 and Y436 in PB1 of raccoon dog viruses (Table 2), suggesting that the raccoon dog viruses may be highly virulent in mice and ducks.

**Table 2:** Amino acid comparison of HA, NA, PB2, PB1, and NS of raccoon dog/SD/sd1/2005 and reference viruses.

Virus	PB2	PB1		HA^a^			NA			NS1	
	627	13	436	133	226	228	49–68	274^b^	294^b^	80–40	PDZ-binding domain
Raccoon dog/SD/sd1/2005	E	P	Y	S	Q	G	−	H	N	−	ESEV
BHgoose/QH/0510/05	K	P	Y	S	Q	G	−	H	N	−	ESKV
MDK/JX/2300/2005	E	P	Y	S	Q	G	−	H	N	−	ESEV
CK/ST/4231/2003	E	P	Y	S	Q	G	−	H	N	−	ESEV
SW/SD/2/03	E	P	Y	S	Q	G	−	H	N	+	EPEV
DK/SD/093/2004	E	P	Y	S	Q	G	−	H	N	+	ESEV
VN/1194/2004	K	P	Y	L	Q	G	−	H	N	−	ESEV
BJ/01/2003	E	P	Y	S	Q	G	−	H	N	−	ESEV
HK/213/03	E	P	Y	L	Q	G	+	H	N	−	ESEV
HK/156/97	E	P	Y	S	Q	G	−	H	N	+	EPEV
GS/GD/1/96	E	P	Y	S	Q	G	+	H	N	+	ESEV

a H3 numbering.

b N2 numbering.

+ No amino acid deletion.

− Amino acid deletion.

NS1 protein plays an important role in the pathogenicity of H5N1 virus in different hosts. A five amino acid residue deletion at positions 80 to 84 was found in the NS1 molecule of the raccoon dog viruses (Table 2) as also characterized for the other H5N1 viruses of the Z+, Z, Y, A, B and C genotypes, and this deletion may contribute to increased virulence [44]. Previous studies showed that several mutations, such as P42S, D92E, and V149A, may increase the virulence of H5N1 viruses in pigs, mice and chickens, respectively [45], [46]. Zhu et al showed that a deletion of amino acids 191 to 195 of the NS1 protein could contribute to the attenuation of influenza A virus in chickens [20]. This was not observed in raccoon dog viruses. Large-scale sequence analysis of avian influenza viruses indicated that the four C-terminal residues of the NS1 protein is a potential PDZ ligand binding motif of the X-S/T-X-V type [47]. The PDZ ligand binding motifs with the sequence of ESEV or EPEV were found in the NS1 from the highly pathogenic H5N1 viruses isolated in 1997 and 2003 as well as the 1918 pandemic virus (all of avian origin). They are able to bind cellular PDZ-containing proteins involved in host cellular signaling pathways. In contrast, the NS1 protein in most low pathogenic human influenza viruses contain a different motif (RSKV or RSEV), which cannot bind PDZ-containing proteins. A recent study showed that the PDZ-binding motif of NS1 is a new virulence factor of influenza A viruses [48]. Raccoon dog viruses possessed an “avian-like” ESEV motif at the NS1 C-terminal region, PDZ domain motif, which may contribute to increased virulence (Table 2).

## Discussion

HPAI H5N1 viruses have become endemic in poultry in southern China and southeast Asia since 2003. Genetic analysis revealed that this epidemiology resulted in the establishment of multiple different H5N1 sublineages [6], [8], [10]. Due to continued genetic reassortment, a diversity of genotypes of H5N1 virus was detected in 2001 and 2002 [8]. By 2003, genotype Z began to emerge as a dominant virus in southern China, and has spread to southeast Asia, central Asia, the Middle East, Europe, and Africa [1], [9]. In contrast, H5N1 viruses introduced into Japan and South Korea in 2003–2004 belonged to genotype V [49]. Genotype V virus was first isolated from chickens in Southern China in 2003, and was then continuously found in wild birds in 2005–2006 [6]. Interestingly, genotype V viruses may have contributed the HA, NA, and NP genes to QH-like viruses [1], [6]. The H5N1 viruses recently isolated in human and mammalian animals were genotype Z, except for the H5N1 viruses found in viverrids in Vietnam (genotype G) [1], [19]. In this study, the raccoon dog viruses genetically belong to genotype V. To our knowledge, this is the first report that a genotype V H5N1 virus is associated with the infection of mammals.

The pathogenicity of H5N1 influenza virus is polygenic, at least including HA, NA, PB2, PB1, PA, NP and NS genes [1]. Many studies indicate that pathogenicity depends on the functional integrity of each gene and on a cluster of genes that is optimal for infection [24], [39], [41], [50]. Recent studies have revealed that the high pathogenicity of H5N1 virus is a complex phenotype dependent on both the virus and the host [24], [41]. HA plays an important role in determining the tissue tropism, systemic spread, and pathogenicity of avian influenza viruses. Influenza viruses attach to host cells by binding of the hemagglutinin to sialosaccharides on the host cell surface. Receptor binding preference is a major factor in determining host species tropism. Human influenza viruses prefer sialic acid (SA)–α-2,6-Gal–terminated saccharides, whereas avian influenza viruses prefer those terminating in SA-α-2,3-Gal [1]. In our study, the HA molecules of the raccoon dog viruses retained the 2,3-NeuAcGal linkage properties, which share the same amino acids Q226 and G228 as avian viruses. The multiple basic amino acids at the HA cleavage site are essential for lethal infection in chicken and mouse, and the raccoon dog viruses contain a multibasic cleavage site in the HA. These results are consistent with the observations that these raccoon dog viral isolates are highly pathogenic to chickens.

A balance between the activity of HA in virus attachment and that of NA in virus release is crucial for optimal viral replication [50]. Similar to the other genotype V and Z viruses, the raccoon dog viruses also exhibited a deletion in the stalk region of NA, which is proposed to be an adaptation of H5N1 viruses from aquatic birds to terrestrial poultry, such as chickens [1]. Using reverse genetics, many studies revealed that some amino acid substitutions of PB2, PB1, PA, and NS1 were associated with H5N1 pathogenicity in mammals [40], [42], [43], [45], [46]. In our study, the raccoon dog viruses have not acquired the 627 L amino acid substitution in PB2, which is an important virulence marker for the infection of mammals. The virus isolates from raccoon dogs contain amino acid residues P13 and Y436 in PB1, which may contribute to the pathogenicity in mice and ducks [43]. However, the molecular pathogenicity of the raccoon dog viruses in mammals remains to be studied by reverse genetics.

Some carnivorous animals, such as tigers, leopards, domestic cats, pet dogs, and viverrids, are naturally infected with H5N1 virus, leading to death [15]–[19]. Dogs experimentally inoculated with H5N1 virus have been shown to be susceptible to infection and can shed virus without apparent disease signs, and the virus can attach to the higher and lower respiratory tract tissues [51], [52]. In contrast, in humans, cats and ferrets, H5N1 virus predominantly attaches to the lower respiratory tract where the SA2,3 Gal receptor is present. This may be a limiting factor in human-to-human transmissibility of H5N1 virus [33], [53]–[55]. The transmission of H5N1 virus to mammalian species is of great concern as it may allow the virus to adapt to mammalian hosts and acquire pandemic potential. Our results of lethal H5N1 infection in raccoon dog extend the range of known mammalian hosts and emphasizes the importance of monitoring in canivorous animals during H5N1 outbreaks.

In this study, the raccoon dogs were caged in a farm, and had no direct contact with chickens other than eating the carcasses. These observations suggest that the source of the raccoon dog infection may come from chicken carcasses. A retrospective epidemiology survey showed that chicken death was observed in several houseback farms at that time (data not shown). The exact source of these viruses remains unknown due to the inability to identify or isolate a genetically related virus at the outbreak region during that time. These results also emphasize the importance of enhanced biosecurity for endangered animals in the epidemiology of an H5N1 influenza virus outbreak.

## Materials and Methods

### Virus isolation and identification

In early January 2005, we collected lung samples from two raccoon dogs (*Nyctereutes procyonoides*) that died of respiratory disease from a farm with 1000 raccoon dogs used for pelts in the Shandong province in eastern China. Subsequent epidemiological investigation revealed that the raccoon dogs had been fed with chicken carcasses, and about 100 died from respiratory disease and/or diarrhea during the month of January, 2005.

10% [w/v] tissue homogenates of the specimens (0.2 ml per egg) were inoculated allantoically in 9-day-old specific-pathogen-free (SPF) embryonated chicken eggs. After incubation at 35°C for 72 h., the allantoic fluids were then harvested and tested for hemagglutinin (HA) activity with a 0.5% suspension of chicken erythrocytes. The isolates were subtyped by hemagglutinin inhibition (HI) and neuraminidase inhibition (NI) assays .Virus-containing allantoic fluid was stored at −80°C. The infectivity of stock viruses was determined in 10-day old embryonated chicken eggs by the method of Reed and Muench and expressed as the log10 50% egg infective dose (EID_50_)/mL of allantoic fluid.

All the experiments using live and infectious viruses were performed in a certified biosafety level 3 laboratory (BSL-3) containment facility, and all personnel were required to use respiratory protection when working with live viruses or experimentally infected animals.

### RT-PCR amplification and genomic sequencing

Viral RNAs were extracted from infectious allantoic fluid by the use of Trizol LS reagent (Invitrogen) as specified by the manufacturer. Uni12 primer was used for reverse transcription. PCR was performed with a set of primers specific for each gene segment of influenza A virus [29]. PCR products were purified with the TaKaRa agarose gel DNA purification kit (TaKaRa). Sequencing was performed in Shanghai Sangon Biological Engineering Technology&Services Co., Ltd (Shanghai, China). Sequences were compiled with the Lasergene sequence analysis software package (DNAStar, Madison, WI, USA).

### Phylogenetic analysis

Nucleotide BLASTn analysis (http://www.ncbi.nlm.nih.gov/BLAST) was used to identify related reference viruses, and the reference sequences were obtained from GenBank. The nucleotide sequences were compared initially using the Megalign program (DNASTAR). Pair-wise sequence alignments were also performed with the Megalign program to determine nucleotide and amino acid sequence similarities. To understand the evolutionary characterization of H5N1 viruses isolated in this study, phylogenetic analyses of the aligned sequences for 8 gene segments were performed by the neighbor-joining method with 100 bootstraps by using PHYLIP (the PHYLogeny Inference Package) version 3.67 (http://evolution.gs.washington.edu/phylip.html). Alignments of each influenza virus sequence were generated using program ClustalX 1.83.

### Pathogenicity experiments in chickens

Pathogenicity tests were performed in accordance with instructions in the World Organization for Animal Health manual. Eight 5-week-old specific-pathogen-free (SPF) chickens were inoculated intravenously with 0.2 ml of a 1∶10 dilution of bacterium-free allantoic fluid of the isolates with 10^−8.4^ 50% egg infective doses (EID50)/mL to determine the intravenous virus pathogenicity index (IVPI).
